# Hughes-Stovin Syndrome: A Case Report on a Rare, Life-Threatening Vasculitis

**DOI:** 10.31138/mjr.290823.hsr

**Published:** 2023-08-29

**Authors:** Tayyeba Khursheed, Ahmed Masood, Muhammad Sufyan Khan, Muhammad Sharif, Somaya Shah, Muhammad Arqam Miraj

**Affiliations:** Department of Rheumatology, Pakistan Institute of Medical Sciences, Islamabad, Pakistan

**Keywords:** vasculitis, Behçet’s disease, rare diseases, aneurysm

## Abstract

Hughes-Stovin Syndrome (HSS) is a rare vasculitic disorder characterised by widespread pulmonary artery aneurysms. It shares some features with Behçet disease. Currently, the diagnosis is based on clinical suspicion. Our case describes a young male who presented with haemoptysis and previous history of pulmonary embolism. Workup was essentially unremarkable, but imaging revealed multiple pulmonary artery aneurysms. Timely initiation of glucocorticoids and immunosuppression with cyclophosphamide led to improvement. High-dose glucocorticoids and immunosuppressants are the mainstays of treatment. Untreated cases can result in fatal outcomes.

## INTRODUCTION

Hughes-Stovin Syndrome (HSS) is a rare vasculitic disorder affecting peripheral and pulmonary vasculature. HSS is characterised by widespread pulmonary artery aneurysms (PAA). It was first described in 1959 by English physicians Hughes and Stovin, thus named HSS.^1^ The patients had evidence of systemic disease, a history of recurrent venous thromboembolism, and autopsy-confirmed pulmonary artery aneurysms. Later, several cases published in the literature described similar presentations in young patients.^2–5^ HSS has received particular attention in the recent past with the formation of a multi-national and multidisciplinary HSS international study group.^6–7^ HSS classically manifests with recurrent deep venous thromboses, venous and arterial thrombosis with or without pulmonary artery aneurysms, and recurrent thrombophlebitis.^6^ Patients may also have oral or genital ulcers and other features of Behçet’s Disease (BD),^6^ and is considered a variant of BD by some clinicians.^8–9^ There are no diagnostic or classification criteria for HSS. Diagnosis is purely on a clinical basis in a patient with recurrent unexplained arterial and venous thromboembolism with a normal coagulation profile. Imaging helps aid diagnosis, particularly with a demonstration of PAA on computed tomography pulmonary angiogram (CTPA).

## PATIENT INFORMATION

A 30-year-old Pakistani male, with a history of smoking marijuana and unprovoked pulmonary embolism, presented to the emergency for evaluation. A year before his presentation, he presented at another hospital with dyspnoea and haemoptysis. He was worked up at that time and diagnosed with pulmonary embolism based on radiographic findings, without any evidence of pulmonary aneurysms. He was started on Rivaroxaban which he discontinued seven months later. His family history was unremarkable, and he did not have any risk factors for venous thromboembolism (VTE).

## CASE PRESENTATION

At the presentation, he complained of chest pain, haemoptysis, and dyspnoea. On examination, he appeared tachypnoeic but was maintaining oxygen saturation. The general and systemic examination was unremarkable. In addition, there was no evidence of deep venous thrombosis on doppler evaluation of lower limbs. Complete blood count and metabolic workup were unremarkable. ESR was mildly elevated at 45mm/hour. The coagulation profile was within the normal range, and a thrombophilia screen was negative. In addition, Hepatic B, Hepatitis C, and Tuberculosis screening were negative. A summary of the investigation results from the patient is available in **Table 1**.

**Table 1.** Summary of investigations carried out in the patient.

**Test**	**Result**	**Reference Range**
*Complete Blood Count*		
White Blood Count	9870	4000–11,000/μL
Haemoglobin	13.4	11–18g/dL
Platelet Count	357,000	150,000–450,000 μL
*ESR*	45	0–10mm/hour
*Liver Function Tests*		
Bilirubin (Total)	0.3	0.3–1.2mg/dL
ALT	39	4–42 U/L
Alkaline Phosphatase	135	40–130 U/L
*Coagulation Screen*		
PT	13	10–14 seconds
APTT	17.64	28–42 seconds
*Renal Function Tests*		
Urea	41	13–43 mg/dL
Creatinine	0.9	0.7–1.3 mEq/L
*Urine Analysis*		
Protein	Nil	Negative
Red blood cells	Nil	Negative
Casts	Nil	Negative
*Sputum Analysis*		
Acid Fast Bacilli	Negative	Negative
Xpert MTB	Negative	Negative
*Thrombophilia Screen*		
Factor V Leiden	Negative	Negative
Prothrombin Gene Mutation	Negative	Negative
Antithrombin III	Normal	80–120%
Protein C	Negative	70–140%
Protein S	Negative	60–140%
Lupus Anticoagulant	Negative	Negative
Anticardiolipin	Negative	Negative
Beta-2 Glycoprotein	Negative	Negative
*Autoantibodies*		
Anti-Nuclear Antibodies	Negative	<1:40
Anti-Double Stranded DNA Antibodies	Negative	<30 IU
Anti-Neutrophilic Cytoplasmic Antibodies	Negative	<1:20
Anti-Glomerular Basement Antibodies	Negative	<20U/L
*Viral Hepatitis Screen*		
Anti-HCV	Negative	Negative
HBs Ag	Negative	Negative

A plain chest radiograph showed a lobular opacity on the left hilum. CTPA showed multiple areas of focal dilatation within segmental branches of the left pulmonary arterial tree representing uni-lateral peripheral pulmonary artery aneurysms with adjacent focal soft-tissue attenuation suggestive of perianeurysmal inflammation or mural thrombus. Other findings of note included subpleural linear fibrotic bands but no evidence of current pulmonary embolism.

The patient was referred to the rheumatology team. Upon evaluation, the patient described a history of oral and genital ulcers. Inspecting the oral cavity and genitalia confirmed scarring from the previous ulceration. A skin pathergy test was performed, which was negative. There were no signs of eye disease, and the slit-lamp examination was unremarkable.

### Clinical Management

The patient was given a pulse of 1g IV Methylprednisolone followed by 1mg/kg of oral prednisolone, which was gradually tapered each visit. He was also started on 1g IV cyclophosphamide monthly for six months.

### Follow-up

After six months, the patient did not develop a new episode of haemoptysis. CTPA was repeated, which showed the persistence of old aneurysms but no new aneurysm formation or radiographic progression.

## DISCUSSION

We treated a case of HSS who presented with haemoptysis and PAA. Our patient responded to corticosteroid and cyclophosphamide therapy and did not develop deterioration or progression on follow-up. PAA can be secondary to various causes, broadly classified into congenital and acquired causes. Eisenmenger’s syndrome and certain heritable connective tissue diseases such as Ehlers-Danlos and Marfan syndrome are associated with PAA. Pulmonary hypertension, autoimmune diseases, and vasculitis are important causes of acquired PAA. Other causes, including trauma and iatrogenic factors, can lead to the formation of pseudoaneurysms.^10^

**Figure 1.**
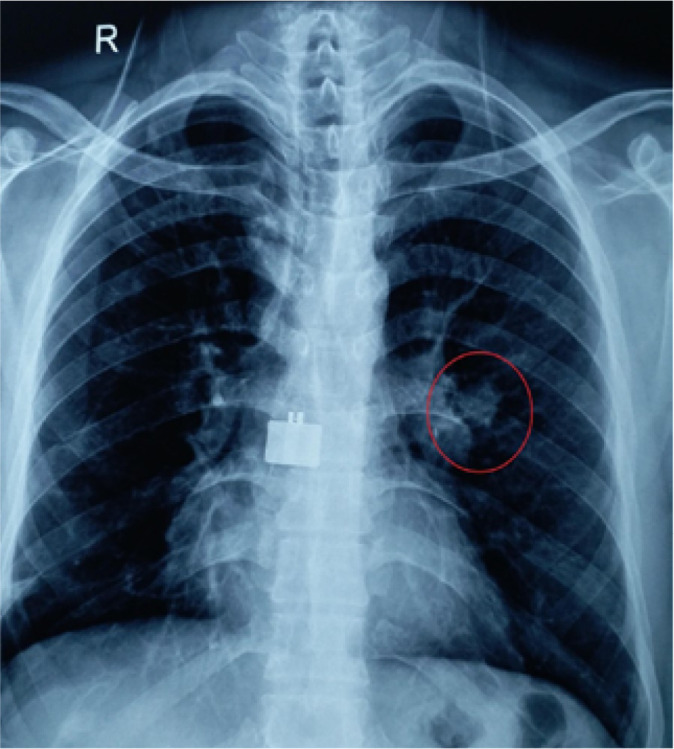
Radiographic presentation of Hughes-Stovin Syndrome. Pulmonary artery aneurysm giving the impression of a nodular opacity on chest x-ray.

**Figure 2.**
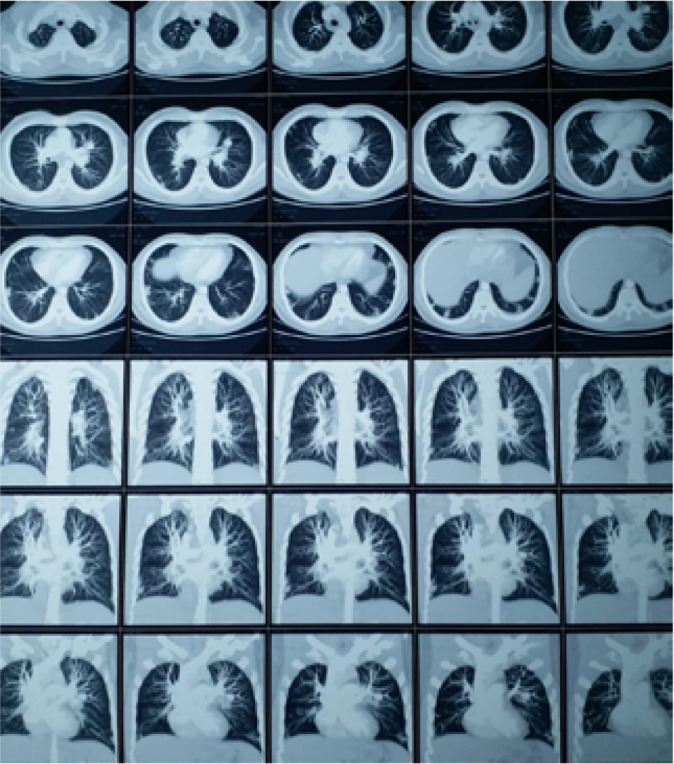
Radiographic presentation of Hughes-Stovin Syndrome. CT-Pulmonary Angiogram revealing pulmonary artery aneurysms.

Our patient had none of the features associated with the above-mentioned conditions; given the history of the recurrent unexplained thromboembolic phenomenon and some features of BD, a diagnosis of HSS was made. Whether HSS is part of the spectrum of BD or an independent disease has been debatable. However, overlapping features with BD have led many to believe it is a subtype or incomplete form of BD.^8–9^ The new international criteria for BD is based on a sign and symptom scoring system where ocular lesions, genital aphthosis, and oral aphthosis carry two-point each. Skin lesions, neurological manifestations, and vascular manifestations take one point each. A positive pathergy test has a score of one and is an optional criterion. A score of 4 or more indicates BD.^11^ **Table 2** summarises prominent clinical features, treatment used, and outcomes of selected case reports from the literature.

**Table 2.** A tabular presentation of similar case reports from the literature review^1–6^

**Author**	**Year**	**Age**	**Sex**	**Clinical Features**	**Treatment**	**Outcome**
Hughes JP, Stovin PG	1958	35	Male	Haemoptysis Recurrent VTE Pulmonary artery aneurysms (PAA)	Anticoagulation (initially) Lobectomy	Death from massive haemoptysis
	1958	14	Male	Otitis media Recurrent VTE Haemoptysis PAA	Right subtemporal decompression Posterior tibial artery ligation Cortisone	Death from massive haemoptysis
Kopp WL, Green RA	1962	30	Male	Periodic fever Recurrent VTE Haemoptysis PAA	Anticoagulation (initially) Antibiotics Exploratory thoracotomy Prednisone	Death from massive haemoptysis
Kirk GM, Seal RM	1964	29	Male	Periodic fever Recurrent VTE Haemoptysis PAA	Prednisone Anticoagulation Thoracotomy	Unknown
George Teplick J et al.	1974	25	Female	Fever Haemoptysis Recurrent VTE PAA	Antibiotics	Death from massive haemoptysis
Kinjo M et al.	1978	37	Female	Recurrent VTE Haemoptysis PAA	Antitubercular therapy Left lower lobectomy Corticosteroids	Death from massive haemoptysis
Tsai CL et al.	2005	34	Male	Haemoptysis PAA	Corticosteroids	Improvement
Emad Y et al.	2007	26	Male	DVT Haemoptysis PAA	Anticoagulation (initially) Corticosteroids Cyclophosphamide	Improvement
		16	Male	Recurrent VTE PAA	Anticoagulation Corticosteroids Azathioprine	Improvement
Kechida M et al.	2017	55		DVT Recurrent orogenital ulcers PAA	Anticoagulation Corticosteroids Cyclophosphamide Azathioprine	Improvement
El Jammal T et al.	2018	19	Male	Fever Recurrent VTE Haemoptysis	Anticoagulation Corticosteroids Cyclophosphamide	Improvement
Emad Y et al.	2019	35	Male	Recurrent VTE	Anticoagulation Corticosteroids Cyclophosphamide	Improvement

The management of HSS is quite similar to BD. An initial pulsed Methylprednisolone followed by a maintenance regime of glucocorticoid is the most common initial treatment option. Various immunosuppressant and biologic options have been tried for steroid-sparing with varying success. The most common immunosuppressive agents used to treat HSS include IV cyclophosphamide and azathioprine. Biological drugs from within the class of TNF inhibitors are also frequently used.^6^

Early treatment with glucocorticoids and single or combined immunosuppressive agents is associated with improved outcomes. This is evident from **Table 2**, where outcomes were improved in patients on immunosuppressive therapy compared to earlier cases where treatment was mostly empiric. The incidences of inferior vena cava thromboses and rupture of PAA are associated with poor outcomes. Prompt initiation of glucocorticoids and cyclophosphamide led to improvement in our patient. However, untreated disease can rapidly complicate into fatal consequences. Therefore, all cases should be treated promptly and adequately when suspicion of HSS is made.

## CONCLUSION

HSS is a rare, life-threatening disorder that can affect pulmonary vasculature. When clinically suspected, relevant imaging modalities can aid in diagnosis. The disease has many overlapping features with BD and should be treated similarly. Untreated disease can have fatal outcomes. Prompt treatment with glucocorticoids and immunosuppressants can be life-saving.
